# Twnbiome: a public database of the healthy Taiwanese gut microbiome

**DOI:** 10.1186/s12859-023-05585-6

**Published:** 2023-12-14

**Authors:** Amrita Chattopadhyay, Chien-Yueh Lee, Ya-Chin Lee, Chiang-Lin Liu, Hsin-Kuang Chen, Yung-Hua Li, Liang-Chuan Lai, Mong-Hsun Tsai, Yen-Hsuan Ni, Han-Mo Chiu, Tzu-Pin Lu, Eric Y. Chuang

**Affiliations:** 1 Bioinformatics and Biostatistics Core, Centers of Genomic and Precision Medicine, National Taiwan University, Taipei, Taiwan; 2 Department of Biomedical Engineering, China Medical University, Taichung, Taiwan; 3 Department of Public Health, Institute of Health Data Analytics and Statistics, National Taiwan University, Taipei, Taiwan; 4 Graduate Institute of Biomedical Electronics and Bioinformatics, National Taiwan University, Taipei, Taiwan; 5 Center for Biotechnology, National Taiwan University, Taipei, Taiwan; 6 Graduate Institute of Physiology, National Taiwan University, Taipei, Taiwan; 7 Institute of Biotechnology, National Taiwan University, Taipei, Taiwan; 8 College of Medicine, National Taiwan University, Taipei, Taiwan; 9 Department of Internal Medicine, National Taiwan University Hospital, Taipei, Taiwan; 10 Institute of Health Data Analytics and Statistics, National Taiwan University, Taipei, Taiwan; 11 Biomedical Technology and Device Research Laboratories, Industrial Technology Research Institute, Hsinchu, Taiwan; 12 Division Research and Development Center for Medical Devices, National Taiwan University, Taipei, Taiwan

**Keywords:** Gut-microbiota, Public database, Taiwan population, Healthy subjects

## Abstract

With new advances in next generation sequencing (NGS) technology at reduced costs, research on bacterial genomes in the environment has become affordable. Compared to traditional methods, NGS provides high-throughput sequencing reads and the ability to identify many species in the microbiome that were previously unknown. Numerous bioinformatics tools and algorithms have been developed to conduct such analyses. However, in order to obtain biologically meaningful results, the researcher must select the proper tools and combine them to construct an efficient pipeline. This complex procedure may include tens of tools, each of which require correct parameter settings. Furthermore, an NGS data analysis involves multiple series of command-line tools and requires extensive computational resources, which imposes a high barrier for biologists and clinicians to conduct NGS analysis and even interpret their own data. Therefore, we established a public gut microbiome database, which we call Twnbiome, created using healthy subjects from Taiwan, with the goal of enabling microbiota research for the Taiwanese population. Twnbiome provides users with a baseline gut microbiome panel from a healthy Taiwanese cohort, which can be utilized as a reference for conducting case-control studies for a variety of diseases. It is an interactive, informative, and user-friendly database. Twnbiome additionally offers an analysis pipeline, where users can upload their data and download analyzed results. Twnbiome offers an online database which non-bioinformatics users such as clinicians and doctors can not only utilize to access a control set of data, but also analyze raw data with a few easy clicks. All results are customizable with ready-made plots and easily downloadable tables. Database URL: http://twnbiome.cgm.ntu.edu.tw/.

## Background

With the recent advancements in next generation sequencing (NGS) technology, the study of microbiota has been growing rapidly [1–4]. Traditionally, microbial genomics utilized cultivation-based methods to study genomes of microbes in the environment [5]. However, such traditional methods miss out on capturing much of the microbial diversity, as it is challenging to culture large groups of microorganisms properly. With the advent of 16S ribosomal RNA (16S rRNA) sequencing techniques, microbiota-related research, databases, and publications have all been growing exponentially [6]. Moreover, since 2008, with the initiation of the Human Microbiome Project (HMP), which has already released more than 2,200 microbial genome sequences isolated from various human body sites, including the feces, nasal cavity, throat, gut, and so on (https://portal.hmpdacc.org), microbiome research has undergone immense and rapid development [7].

Among all sites, the gut microbiota from the gastrointestinal tract are considered to be one of the biggest ensembles of microbial flora in the human body. The adult human gut contains about 10^14^ bacterial cells from more than 1,000 different bacterial species, which are involved in numerous human metabolic and physiological functions [8–10]. The host benefits from the homeostatic balance provided by the bacteria; however, a change in the microbial composition can lead to a severe imbalance between the beneficial and potentially pathogenic bacteria, thus making the gut vulnerable to microbial alterations. This imbalance, also known as “dysbiosis”, can cause physical symptoms or even diseases [11, 12], which have spawned a plethora of research endeavors towards unraveling the relationship between different diseases and microbiota [13–16]. Such studies aimed to provide descriptions of characteristic alterations in the composition of the microbiome that may prove useful as diagnostic biomarkers [17]. Furthermore, to elucidate the therapeutic potential of the human gut microbiome through manipulation, administration of a solution of fecal matter from a donor into the intestinal tract of a recipient has been used to directly change the recipient’s microbial composition in attempts to confer a health benefit [18–20]. This procedure, known as “fecal microbiota transplantation”, has already been extrapolated from animal models to human beings and has been used to successfully treat recurrent *Clostridium difficile* infection [21]. Preliminary findings indicate that it may also carry therapeutic potential for other conditions such as inflammatory bowel disease, obesity, metabolic syndrome, and functional gastrointestinal disorders. Therefore, microbiota have implications for human health, making them potential biomarkers for clinical applications.

It is well-known that the microbiome is diverse and heterogeneous in different individuals, even in the same tissue type. Several important factors, such as diet, disease phenotype, and environmental exposures, can have great impact on the abundance and composition of the microbiome [22, 23]. According to a recent study, the geographical location of the recruited subjects had a greater effect on human gut microbiota variations than disease phenotype in the constructed model [24]. This suggests that cross-national studies of potential microbiota biomarkers is not a good strategy. Therefore, it becomes challenging to identify consistent signatures as the geographic scale becomes larger, making it more difficult to establish a standard or a normal reference baseline that would be applicable globally [25]. Hence, as the microbiome segregated across ethnic and geographic populations could potentially lead to differences in disease severity [26, 27], developing population-specific databases containing the microbiome data from healthy individuals is a priority.

Furthermore, combining the demand for fast computation speed and large data storage, NGS analyses are mostly performed remotely on powerful servers using command-line interfaces. Therefore, the tools involved are often developed without a graphical user interface (GUI) to maximize computation efficiency. This kind of terminal-based environment imposes a considerable barrier, especially for biologists and clinicians not trained in bioinformatics. Furthermore, the output of most analysis tools is log files or other file formats that are friendly for computers to parse but may not be easily interpreted by humans without data cleaning and transformation. Therefore, for convenience of access and interpretation, to facilitate specific queries by users without advanced bioinformatics skills, creating a user-friendly database from metagenome resources is a current need in the field of microbiome research.

In this study we established the first public gut microbiota database, from healthy subjects in Taiwan, titled Twnbiome (http://Twnbiome.cgm.ntu.edu.tw), that enables microbiome research for populations in Taiwan. Researchers can easily access and compare the composition of microbiota from diseased subjects using Twnbiome as the healthy reference dataset. Twnbiome further provides a 16S rRNA analysis pipeline and statistically comparable metrics with a GUI, for easy access and convenient interpretation.

## Construction and content

### Database overview

**Fig. 1:**
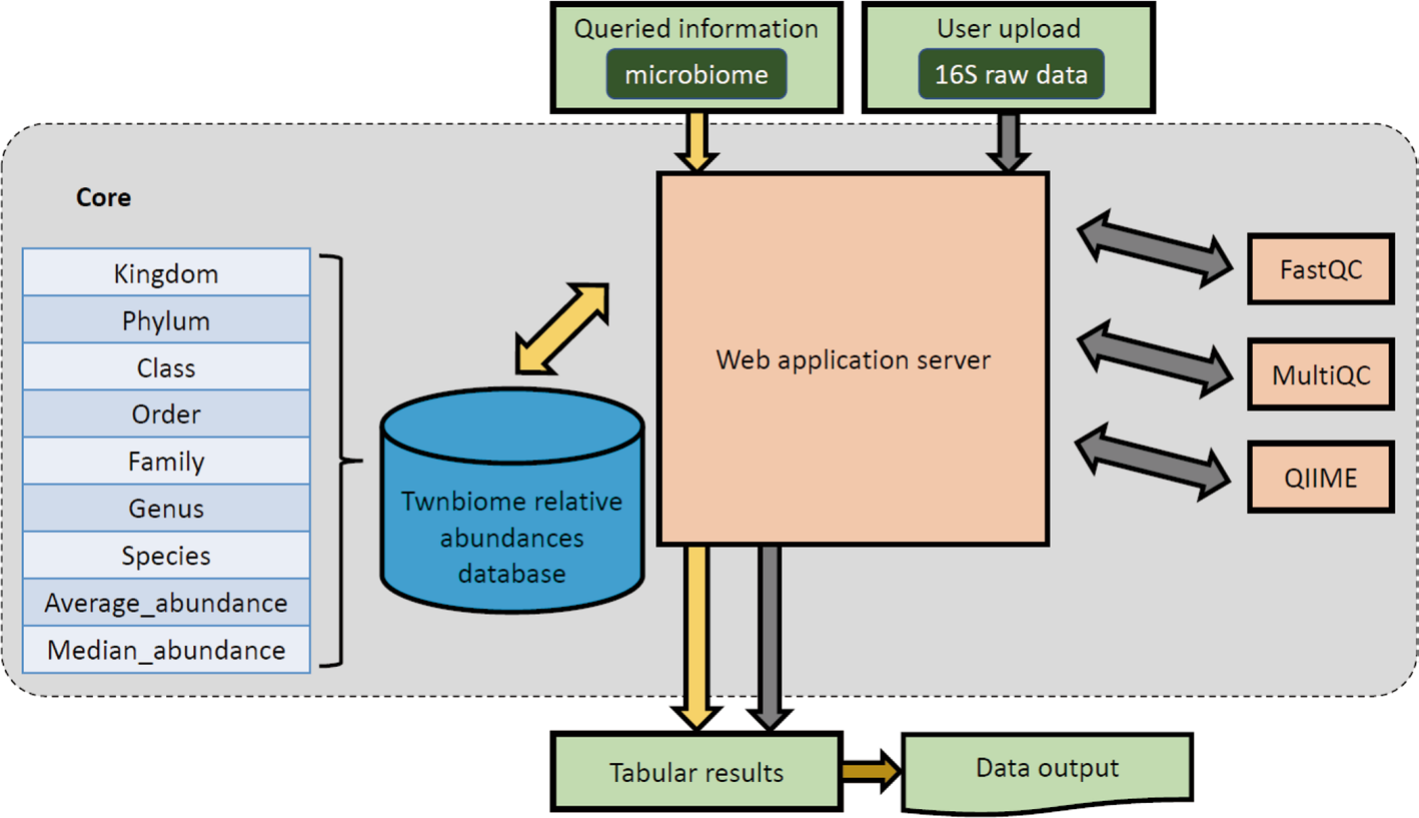
Overview of the Twnbiome database

**Fig. 2:**
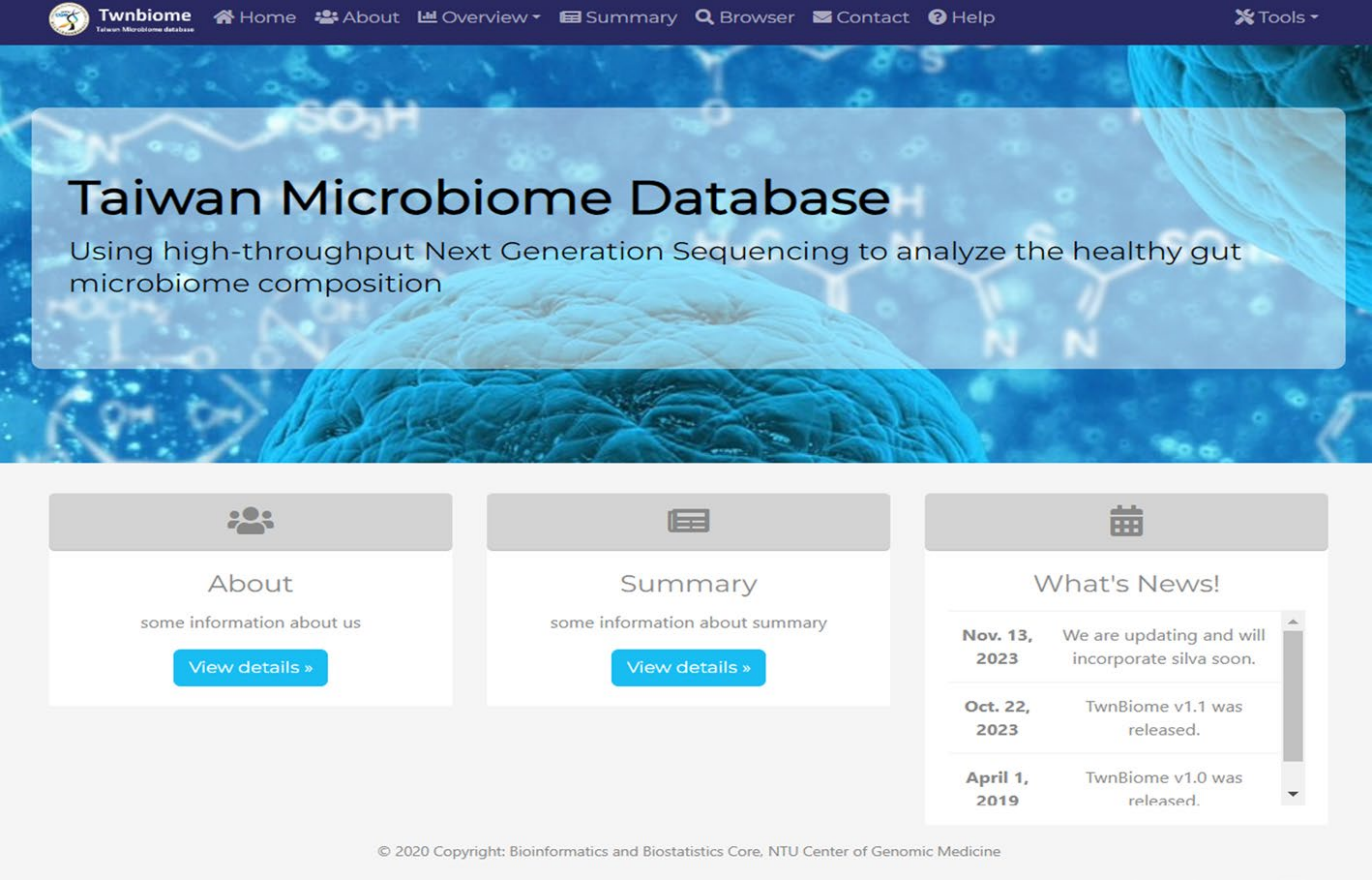
Screenshot of the welcome page of the Twnbiome database

Figure 1 provides an overview of the Twnbiome database. The database primarily offers two main utilities: (i) Twnbiome healthy baseline and (ii) User uploaded data analysis. The Twnbiome database provides users with information regarding the microbiota composition of healthy Taiwanese subjects. The users can further explore all information under different classifications such as gender, age, body mass index (BMI), and so on (Table 1), through 3 different functions: (a) overview, (b) summary, and (c) browser (Fig. 2). The ‘Data Analysis’ utility requires users to upload their 16S ribosomal RNA (rRNA) sequence data, where the built-in bioinformatics pipeline is utilized to conduct analysis. Twnbiome was developed with Django 2.2 and runs on Python 3.6.7 and MySQL 5.7.29.

**Table 1:** Demographic characteristics of 119 healthy Taiwanese subjects in Twnbiome

	Healthy Subjects (N = 119)
*Gender*	
Male	38 (31.93)
Female	81 (68.07)
* Age*	
Adult (18 to 44)	55 (46.21)
Middle Age (45 to 64)	45 (37.82)
Old Age (65 to 94)	19 (15.97)
* BMI*	
Normal (18.5 to < 24)	81 (68.07)
Overweight (24 to < 27)	38 (31.93)
Physical activity	
Sedentary	22 (18.49)
Light	38 (31.93)
Moderate	54 (45.38)
High	5 (4.20)
* Probiotic/Nutritional supplement intake*	
Used	50 (42.02)
Never used before	69 (57.98)
* Diet type*	
Balanced	71 (59.66)
More vegetables	26 (21.85)
More Meat	18 (15.13)
Vegetarian	4 (3.36)
* Meal timing ontime or not *	
Hardly	2 (1.68)
Sometimes	18 (15.13)
Often	38 (31.93)
Usually	61 (51.26)
*Eating out frequency *	
Never	13 (10.92)
Sometimes	43 (36.14)
Often	49 (41.18)
Usually	14 (11.76)
Lifestyle*	
Normal	49 (41.18)
Abnormal	70 (58.82)

Data are presented as n (%) for each category

*BMI* body mass index

*Lifestyle included sleep/wake time regularity of the recruited subjects were regular or not. If the recruited subject has the regular sleeping and waking-up time, he/she would belong to the normal group and vice versa

### Database content

Twnbiome provides a comprehensive gut microbiome landscape of 119 healthy subjects from Taiwan. The healthy volunteers were recruited via the Health Management Center of National Taiwan University Hospital (NTUH), and all participants provided informed consent during their routine health checkups. Fecal samples were collected from each volunteer and all information from the subjects was recorded by a trained interviewer. The study was approved by the institutional review board of NTUH (#201801085RINB).

#### Subject inclusion and clinical information

Individuals aged 18 years and above, who self-reported as healthy and were of Taiwanese Han Chinese ancestry, were recruited. Clinical information from all subjects was obtained from NTUH. Additionally, each subject was asked to self-report information on their physical activity, meal timings, probiotic/nutritional supplement intake, diet type, frequency of eating out, and sleep/wake regularity (lifestyle), through a customized questionnaire. Participants self-reported as healthy if they did not have any of the following diseases: autoimmune disease (rheumatoid arthritis, systemic lupus erythematosus, ankylosing spondylitis, hyper/hypothyroidism, psoriasis, type I diabetes, and multiple sclerosis), neuropsychiatric disease (mood disorders, schizophrenia, autism spectrum disorder), gastrointestinal disease (diarrhea), metabolic diseases (hypertension, atherosclerosis, type II diabetes, non-alcoholic fatty acid), cancers, or other major illnesses. Other basic demographic information including age, sex, and body mass index (BMI) was reported. Considering the potential effects of BMI on microbiota, subjects with BMI < 18.5 (underweight) or BMI ≥ 27 (obese) were excluded from the study. Lastly, subjects with recent drug usage (less than 3 months), including antibiotics, anti-hypertensives, hypolipidemic agents, steroids, antipsychotic and gastrointestinal drugs, were further excluded. Finally, 213 subjects passed all inclusion criteria, out of which 119 fulfilled the criteria of healthy subjects (Table 1).

#### Biosample collections and 16s rRNA sequencing

One-gram fecal samples were collected with user-friendly sterilized kits, and the sample transition process was performed by transferring the samples to -80 ℃ refrigerators immediately, for undergoing cold-chain transportation. QIAamp DNA Stool Mini Kit (QIAGEN Inc., USA) was used to extract the microbiota DNA via lysis, and purification procedures were conducted following the manufacturer’s protocol. For quality control, all DNA samples were evaluated by NanoDrop Microvolume Spectrophotometers. Sequencing of 16S rRNA was performed with the Illumina HiSeq platform, and library preparation was done according to the manufacturer’s instructions (Illumina, USA). Briefly, 12.5 ng of DNA was used for PCR (polymerase chain reaction) amplification of the V3 and V4 regions of the 16S rRNA gene.

The PCR products were purified with AMPure XP beads (Beckman Coulter, USA) and subjected to a secondary PCR reaction with primers from the Nextera XT Index kit (Illumina, USA) by adding dual indices and Illumina sequencing adapters. The final libraries (~ 630 bp) were purified with AMPure XP beads and sequenced by the Illumina HiSeq machine with paired-end sequencing (2*300 bp).

#### Bioinformatic analysis

Once the 16S rRNA sequencing experiments were complete, FASTQ files were generated by HiSeq Report and sequence quality control was conducted using FastQC software (https://www.bioinformatics.babraham.ac.uk/projects/fastqc/) on the raw FASTQ files. For further analysis, the analysis pipeline in Quantitative Insights Into Microbial Ecology (QIIME, version 1.9.1) was used [28]. First, PEAR (version 0.9.8) merged the paired-end reads [29], where the reads that did not overlap with more than 30 bp were discarded. The merged sequences then underwent the filtering step of QIIME for quality control with the following criteria: (1) maximum 3 consecutive low-quality base calls were allowed before truncating the reads, (2) at least 75% of consecutive high-quality base calls were included in a read, and (3) no ambiguous characters were allowed in the sequence, while setting < Q20 as the low quality threshold. After filtering, the sequences were clustered with USEARCH [30] to identify the operational taxonomic units (OTUs) with 97% similarity to the reference from the Greengenes taxonomic database (May 2013 version, http://greengenes.lbl.gov/). Then the OTUs were classified into different taxonomic levels: kingdom, phylum, class, order, family, genus, and species. The OTU information was then used to calculate the relative abundance and the bacterial diversity using alpha and beta diversity indexes. The alpha diversity was mainly calculated using the observed OTUs, whereas the beta diversity was calculated using two different methods: unweighted and weighted UniFrac. Furthermore, the enterotype was determined by previously established methods [31].

### Features of Twnbiome database

Figure 2 provides a screen shot of the welcome page of the Twnbiome database (http://Twnbiome.cgm.ntu.edu.tw). The content of the database is offered to users through various tabs on the navigation bar. The information includes (1) exclusion criteria of our recruited subjects; (2) the basic quality-control information for the sequencing data, including raw reads, effective reads, and Q30 value; (3) detailed sample information and classifications using pie charts; (4) beta diversity plots; (5) detailed taxonomic composition under different classifications; and (6) browsers for specific samples and taxa searches. Details of the sample exclusion criteria are provided under the “About” tab. The “Overview” tab includes quality control distribution plots and gut microbiota composition pie charts for healthy subjects, with classifications based on gender, age, BMI, physical activity, alpha-diversity phylum level, enterotypes, probiotic and supplement intake, diet type, meal on-time frequency, frequency of eating out, and lifestyle. Each pie chart is coded with an interactive function that allows users to see the exact number of the recruited subjects. The "Overview" section further offers the beta diversity principal co-ordinate analysis (PCoA) plot of the 119 healthy subjects, to visualize similarities or dissimilarities between the samples. The PCoA plots are also interactive, can be rotated or zoomed in/out, and can be modified by adding legends. In the “Summary” section, a detailed taxonomic composition, from phylum to genus, among the groups with different gender or BMI is provided. Users can use the drop-down list to choose the taxa of interest to obtain the detailed composition. Finally, in the “Browser” section, specific taxa searches are offered where the users can directly type the name of the taxa (Kingdom, Phylum, Class, Order, Family, Genus, and Species) that they are interested in to explore the average and median abundance among the healthy subjects.

### 16S rRNA sequencing data upload and analysis

For the advancement of metagenomic research among the Taiwanese population, the database further offers a user-friendly interface in the “Tools” section for users to analyze their own data. In the upload data section, the user is required to upload their FASTQ files, and when the analysis is done, the user receives a link to the results for them to explore and download. All analyses are conducted using the same protocol as described before in the ‘methods’ section.

## Utility and discussion

### Subject demography

Table 1 gives full demographic details of the subjects in the database. The majority of participants were female (68.07%) and the age of the participants ranged between 18 and 94 years, with 55 adults, 45 middle aged (45 to < 65), and 19 old aged (65 to < 95) participants with a mean age of 47.1 years. Participants were further stratified based on their BMI, physical activity, dietary habits, and lifestyle/sleep patterns. A majority of subjects were in the normal range for BMI (68.07%), had never used probiotics/nutritional supplements (57.98%), reported a balanced diet (59.66%) with regular meal times (51.26%), and had abnormal sleep patterns (58.82%).

### Twnbiome database

Twnbiome was created with the expectation of enhancing microbiome research in Taiwan and facilitating the improvement of patients suffering from microbial-related health problems. Background information on the project and the subject inclusion/exclusion criteria are provided in the “About” section of the web site (Fig. 2).

#### Overview section

**Fig. 3:**
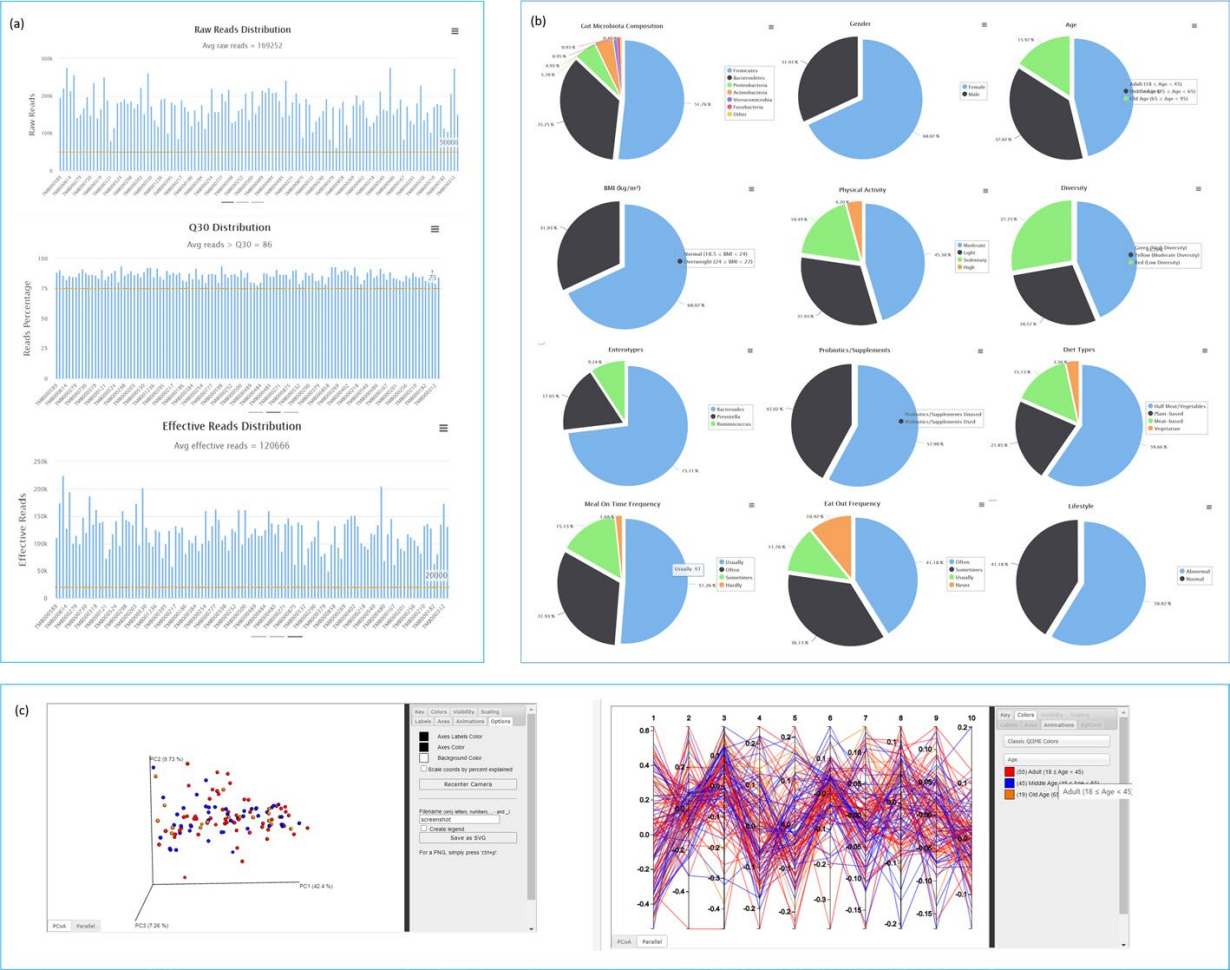
Screenshot of the “Overview” section of the Twnbiome database. **a** Quality plots for the sequencing reads. **b** Pie charts giving a pictorial description of subject-demography under different classifications. **c** Beta diversity plots depicting the amount of species change among subjects with various stratifications

This section covers the quality plots for the sequencing reads (Fig. 3a), pie charts giving a pictorial description of subject demography as described before (Fig. 3b) under different classifications, a sample browser for users to stratify samples based on the demographics and lifestyle factors and beta diversity plots depicting the amount of species change among subjects with various stratifications (Fig. 3c). The average number of raw reads per sample was 169,252, the average Q30 distribution was 87.42%, and the average number of effective reads was 120,666. Overall, the most abundant phylum among healthy Taiwanese subjects was Firmicutes (51.76%) followed by Bacteroidetes (35.25%) (Fig. 3b). The dominating presence of Firmicutes and Bacteroidetes in the gut was consistent with prior findings from studies focusing on healthy microbiome composition [32, 33]. Specifically, when stratified by enterotype composition, it was found that the healthy subjects were segregated into three different enterotypes enriched with Bacteroides, Prevotella, and Ruminococcus (Fig. 3b), again consistent with prior enterotype research findings [34, 35]. Furthermore, the beta diversity PCoA plots for the study subjects displayed no visible significant clusters when stratified by age (Fig. 3c) or any other variable (results not shown), indicating the homogeneity of the healthy subjects that were recruited for this study.

####  Summary section

**Fig. 4:**
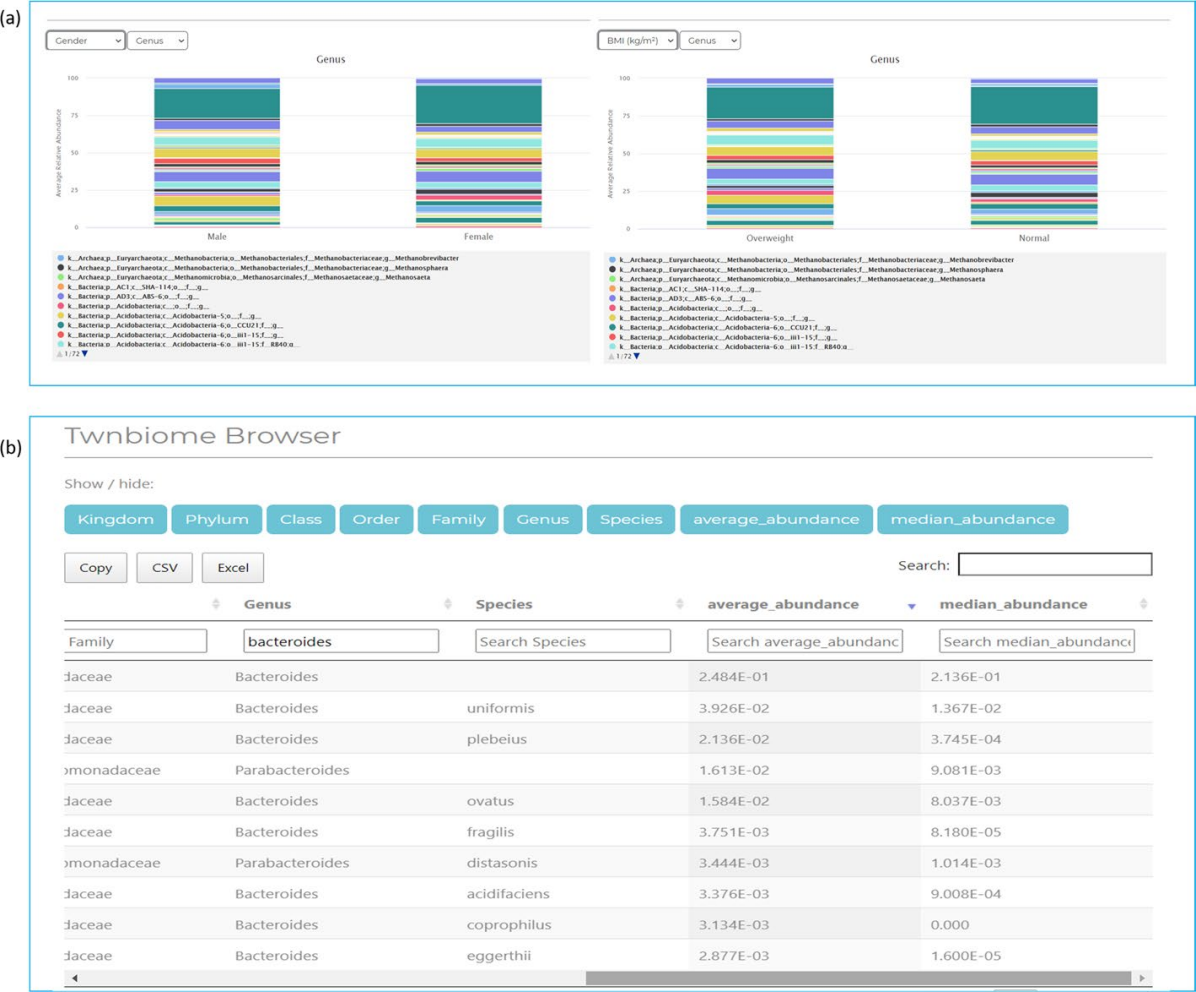
Screen shots of “Summary” and “Browser” sections of the Twnbiome database. **a** “Summary” section showing distribution of microbiome abundance at the genus level for the study subjects when stratified by BMI and sex. **b** “Browser” section, showing taxa with average abundance and median abundance

Figure 4a displays a screenshot of the distribution of microbiome abundance at the genus level for the study subjects when stratified by sex and BMI. These figures can be accessed under the “Summary” section of the Twnbiome web site. The user can similarly select any of the other taxonomic levels (phylum, class, order, or family) to get the distribution of microbiome abundance for both sex and BMI stratification. The user can move the mouse over each of the colored bars to view the name of the corresponding microbiome and its relative abundance. *Bacteroides*, *Faecalbacterium, Acidobacteria, Chlostridiales, Lachnospiraceae, Prevotella, Megamonas, Blautia, Phascolarotobacterium, Megasphaera, Ruminococcus*, and *Bifidobacterium* were among the most abundant genera that were observed in both males/females and normal/overweight groups.

#### Browser section: example 1

Figure 4b provides a screenshot of the “Browser” section of the Twnbiome database. The figure shows the search results for *Bacteroides* at the genus level, ranked by average abundance. The web page displays both average abundance and median abundance for *Bacteroides* as > 20%, consistent with the findings in the “Summary” section, thus making it the most abundant genera among healthy Taiwanese subjects. Furthermore, *Plebeius* and *Uniformis*, as the top abundant species for genus *Bacterioides*, have been found to exist in the guts of healthy Asian individuals and have been extracted for various research purposes in prior studies [36–38].

#### Data analysis: example 2

**Fig. 5:**
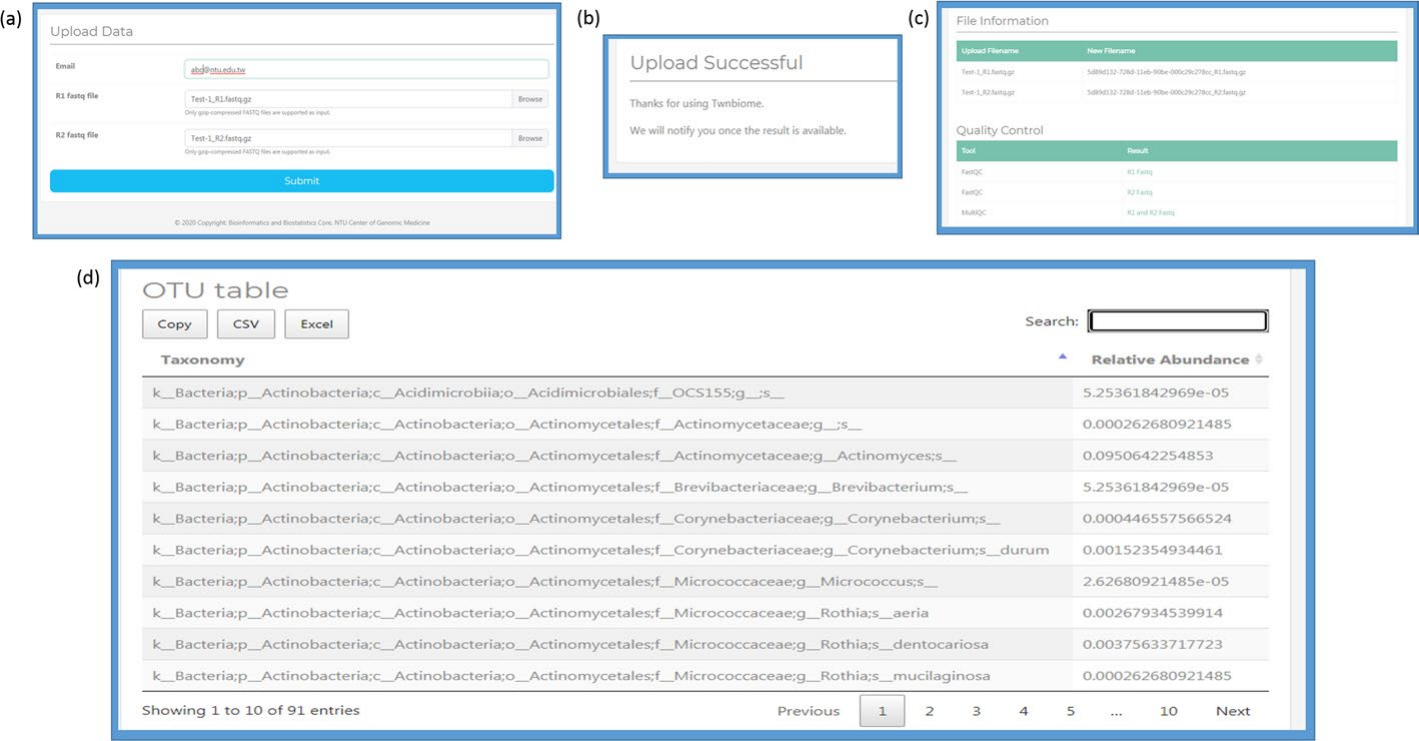
Screen shot displaying an example of the Analysis function of the Twnbiome database. Users can download the results as .xls or .csv tables with a single click. **a** Data upload page where the user is required to upload his/her e-mail address and the paired-end FASTQ files. **b** Screenshot of the page notifying users of the status of their submission. **c** Details of the analysis sent to the users via e-mail. **d** Screenshot of the results: an OTU table with taxonomic information and the corresponding relative abundance

Figure 5 gives an example of the procedure for conducting data analysis on a user-uploaded data, along with the analysis output. The user needs to first click on the “Tools” link at top-right corner of the welcome page (Fig. 2), and will be taken to a data upload page (Fig. 5a) where the user is required to upload an e-mail address and the paired-end FASTQ files. On clicking the submit button, the user will be forwarded to the next page (Fig. 5b), which provides a notification of the status of the submission. Once the analysis is complete the user will receive an e-mail with a link to download the results of the analysis. The results include the information of the data analyzed (Fig. 5c) along with an OTU table containing the taxa information and the corresponding relative abundance (Fig. 5d). Users can download the results as .xls or .csv tables with a single click.

Research on microbiota is on the rise like never before. There are more than 50,000 research articles, just on the gut microbiome, in the Web of Science database since 2000, and as of December 18, 2019, there are already more than 9,500 publications in 2019, a growth of about 30-fold since 2000, sixfold since 2010, and twofold since 2015 [39]. A search in the Web of Science database further reveals a total of 16,716 research articles since 2020 and a total of 5609 publications in 2023 alone (as of 13th November 2023). With such a huge accumulation of research, it is common knowledge that microbiotic composition is affected by many factors, including diet, geographic location, genetics, antibiotics, lifestyle, and so on. Therefore, for conducting microbiome studies under a case-control design, it is very important to have the right comparative groups to explore the potential targets. This along with recent advances in high-throughput sequencing techniques at affordable costs, has led to an enormous amount of gut microbiome data being generated and curated [40]. One significantly important human gut microbiome database is GMrepo [41, 42], which is manually curated from 33 sources with special focus on disease markers. It allows cross-dataset or cross phenotype comparisons of identified markers enabling systematic demonstration of consistent and inconsistent disease-associated microbial markers across datasets. Human gut microbiome atlas (HGMA) (https://www.microbiomeatlas.org/) is another repository that contains health and disease datasets from 20 different countries across five continents and provides region enriched microbial species for different geographical locations. However, both GMrepo and HGMA yet lacks an analysis platform, which can enable users to upload data to conduct metagenomics analyses. Twnbiome is one of the first public databases that shares the microbiota information from more than a hundred healthy Taiwanese individuals and provides population specific enriched species of the gut microbiota. The database, which is still growing, can not only be used to query and obtain summary level data based on phylum, class, order, family, genus, species, but it is the first time that the comprehensive summary statistics of microbiome diversity related to age, sex, lifestyle, and other factors are provided for healthy Taiwanese individuals. Users can further customize the figures and results through interactive platforms for research purposes. It is an unprecedented effort in microbiome research with accurate data acquisition, an established pipeline for data analyses, and the ability to organize, store, access, and share/integrate processed datasets. The release of the database would firmly accelerate microbiota research in Taiwan.

## Conclusion

Twnbiome provides users with a baseline gut microbiome panel from a healthy Taiwanese cohort, which can be utilized as a reference control for conducting case-control studies for a variety of diseases. Twnbiome is an interactive, informative, and user-friendly database that not only provides users with a control dataset, ready to be utilized, but also offers an analysis pipeline, which non-bioinformatics users such as clinicians and doctors can utilize to conduct their analysis with a few clicks, and obtain customizable ready-made plots and easily downloadable tables.

## Data Availability

The database contains all summary statistics and analyzed findings. The raw data can be available on reasonable request from the corresponding author.

## Abbreviations

NGS: Next generation sequencing
rRNA: Ribosomal RNA
HMP: Human microbiome project
GUI: Graphical user interface
NTUH: National Taiwan University Hospital
BMI: Body mass index
QIIME: Quantitative insights into microbial ecology
OTU: Operational taxonomic units
PCoA: Principal co-ordinate analysis
